# Racemic tricarbonyl[(4a,5,6,7,8,8a-η)-2-phenyl-3,4-dihydro-2*H*-1-benzopyran]chromium(0)

**DOI:** 10.1107/S1600536810031028

**Published:** 2010-08-11

**Authors:** Johannes H. van Tonder, Barend C. B. Bezuidenhoudt, J. Marthinus Janse van Rensburg

**Affiliations:** a Department of Chemistry, University of the Free State, PO Box 339, Bloemfontein, 9300, South Africa; b Organic Chemistry, Department of Chemisry, Lund University, PO Box 124, S-221 00 Lund, Sweden

## Abstract

The title compound, [Cr(C_15_H_14_O)(CO)_3_], displays a distorted envelope configuration of the dihydro­pyrane ring. The dihedral angle between the phenyl and phenyl­ene rings is 50.63 (4)°. The Cr^0^ atom is coordinated by three CO groups and the phenyl­ene ring of the flavan ligand in an η^6^ mode, with a common arene-to-metal distance

## Related literature

For general background to chromium(0) complexes of the type [Cr(flav)(CO)_3_] (flav = flavan, flavone or isoflavone ligand), see: Muschalek *et al.* (2007 ▶). For related structures, see: van Tonder *et al.* (2009*a* ▶,*b* ▶). For the synthesis, see: Müller *et al.* (1999 ▶).
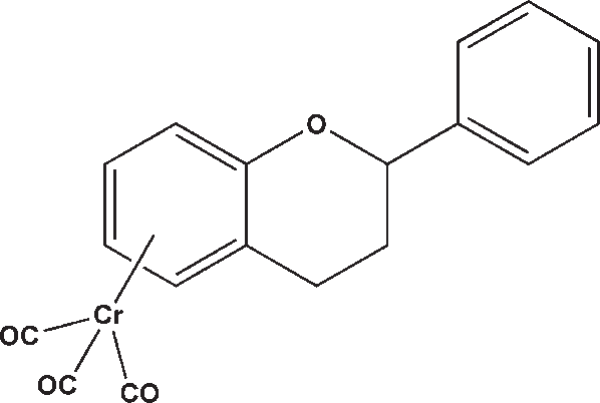


## Experimental

### 

#### Crystal data

[Cr(C_15_H_14_O)(CO)_3_]
*M* *_r_* = 346.29
Monoclinic, 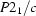

*a* = 12.0275 (2) Å
*b* = 13.1454 (2) Å
*c* = 10.4473 (2) Å
β = 111.717 (1)°
*V* = 1534.55 (5) Å^3^
*Z* = 4
Mo *K*α radiation
μ = 0.76 mm^−1^
*T* = 173 K
0.46 × 0.34 × 0.11 mm

#### Data collection

Bruker APEXII CCD diffractometer
Absorption correction: multi-scan (*SADABS*; Bruker, 2001 ▶) *T*_min_ = 0.766, *T*_max_ = 0.938
20468 measured reflections
5342 independent reflections
3728 reflections with *I* > 2σ(*I*)
*R*_int_ = 0.050

#### Refinement

*R*[*F* ^2^ > 2σ(*F* ^2^)] = 0.038
*wR*(*F* ^2^) = 0.097
*S* = 0.95
5342 reflections
208 parameters
H-atom parameters constrained
Δρ_max_ = 0.67 e Å^−3^
Δρ_min_ = −0.32 e Å^−3^

### 

Data collection: *APEX2* (Bruker, 2007 ▶); cell refinement: *SAINT-Plus* (Bruker, 2007 ▶); data reduction: *SAINT-Plus*; program(s) used to solve structure: *SIR97* (Altomare *et al.*, 1999 ▶); program(s) used to refine structure: *SHELXL97* (Sheldrick, 2008 ▶); molecular graphics: *DIAMOND* (Brandenburg & Putz, 1999 ▶); software used to prepare material for publication: *WinGX* (Farrugia, 1999 ▶).

## Supplementary Material

Crystal structure: contains datablocks global, I. DOI: 10.1107/S1600536810031028/hy2329sup1.cif

Structure factors: contains datablocks I. DOI: 10.1107/S1600536810031028/hy2329Isup2.hkl

Additional supplementary materials:  crystallographic information; 3D view; checkCIF report

## Figures and Tables

**Table 1:** Selected bond lengths (Å)

C5—Cr	2.1936 (18)
C6—Cr	2.2163 (19)
C7—Cr	2.2087 (19)
C8—Cr	2.2251 (17)
C9—Cr	2.2773 (17)
C10—Cr	2.2532 (17)
C11—Cr	1.8353 (19)
C12—Cr	1.8298 (19)
C13—Cr	1.8399 (17)

**Fig. 1.**
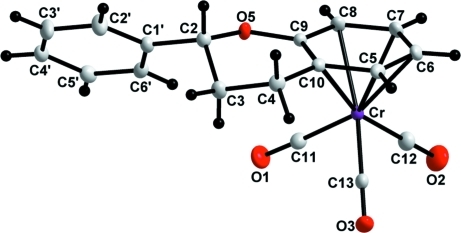
Molecular structure of the title compound. Displacement ellipsoids are drawn at the 30% probability level.

**Fig. 2.**
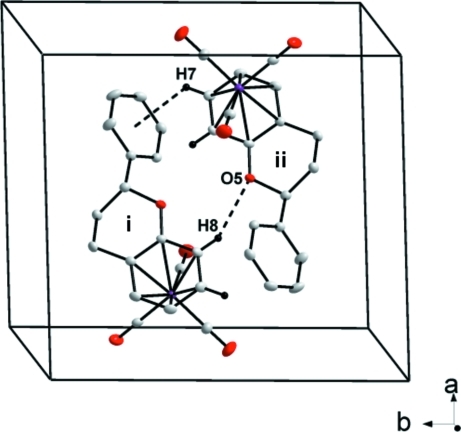
A view of the crystal packing in the unit cell. [Symmetry codes: (i) 1-x, 1/2+y, 1/2-z; (ii) x, 1/2-y, 1/2+z.]

## supplementary crystallographic information

### Comment

The title compound forms part of a series of chromium(0) complexes of the type [Cr(flav)(CO)_3_] (flav = flavan, flavone or isoflavone ligand) (van Tonder *et al.*, 2009*a*,b). Our interest in this type of chromium(0) complexes is partly due to their importance in organic synthesis, as noted earlier (Muschalek *et al.*, 2007).

As illustrated in Fig. 1, the Cr(CO)_3_ group coordinates to the phenylene ring of the flavanoid backbone (Table 1). The distance between the Cr^0^ atom and the centroid of the A-η^6^-coordinated arene ring is 1.731 (1) Å (r.m.s of fitted atoms C5– C10 = 0.0186Å). Molecular distortion is displayed by the twisted flavan backbone. Atoms C4 and O5 is essentially in the plane of the coordinated arene ring (r.m.s of atoms C4–C10 and O5 = 0.0202Å), while atoms C2 and C3 are respectively displaced by -0.244 (2) and 0.478 (2) Å from the plane formed by atoms C4–C10 and O5. This distortion is a result of the expected envelope configuration in the dihydropyran ring. In turn the phenyl ring is twisted away from the plane of atoms C4–C10 and O5, with a dihedral angle of 50.88 (4)°. The crystal packing displays an intermolecular C—H···π soft contact [H···centroid distance = 2.92 Å] between the phenyl ring of one molecule and the arene ring of a neighbouring molecule (Fig. 2). The crystal packing is also stabilized by a C—H···O soft contact between atoms H8 and O5, with a distance of 2.57 Å and a C8—H8···O5 angle of 140°.

### Experimental

Flavan was synthezised via H_2_SO_4_ catalyzed hydrogenation (5 bar) over 10% Pd/C from flavan-4-one [flavan-4-one (2.00 g, 8.9 mmol), 10 % Pd/C (0.10 g), 3 M H_2_SO_4_ (1 ml), EtOH (50 ml)]. Purification by means of flash column-chromatography yielded flavan (0.94 g, 49.9%) as a colourless oil. R_f_ 0.77 (H:A, 8:2).

Preparation of the title compound was based on a method described by Müller et al. (1999). A solution of flavan (0.51 g, 2.4 mmol) and Cr(CO)_6_ (0.53 g, 2.4 mmol) in Bu_2_O:THF (9:1, 10 ml per 100 mg) was degassed with argon, using standard Schlenk techniques, and refluxed (48 h) under an oxygen free atmosphere. The reaction mixture was cooled to room temperature and the solvent evaporated in vacuo. Purification through flash column-chromatography yielded tricarbonyl(A-η^6^flavan)chromium(0) (0.08 g, 9.2%) as a yellow solid. Recrystallization from diethyl ether yielded yellow cuboidal crystals. R_f_ 0.23 (hexane:acetone, 8:2); Mp 170.4°C.

### Refinement

H atoms were positioned geometrically and refined using a riding model, with C—H = 0.93 (aromatic), 0.98 (CH) and 0.97 (CH_2_) Å and with *U*_iso_(H) = 1.2*U*_eq_(C).

### Crystal data

**Table:**

[Cr(C_15_H_14_O)(CO)_3_]	*F*(000) = 712
*M**_r_* = 346.29	*D*_x_ = 1.499 Mg m^−^^3^
Monoclinic, *P*2_1_/*c*	Mo *K*α radiation, λ = 0.71069 Å
Hall symbol: -P 2ybc	Cell parameters from 4557 reflections
*a* = 12.0275 (2) Å	θ = 2.4–30.0°
*b* = 13.1454 (2) Å	µ = 0.76 mm^−^^1^
*c* = 10.4473 (2) Å	*T* = 173 K
β = 111.717 (1)°	Plate, yellow
*V* = 1534.55 (5) Å^3^	0.46 × 0.34 × 0.11 mm
*Z* = 4	

### Data collection

**Table:**

Bruker APEXII CCD diffractometer	3728 reflections with *I* > 2σ(*I*)
graphite	*R*_int_ = 0.050
φ and ω scans	θ_max_ = 32.1°, θ_min_ = 1.8°
Absorption correction: multi-scan (*SADABS*; Bruker, 2001)	*h* = −17→14
*T*_min_ = 0.766, *T*_max_ = 0.938	*k* = −19→19
20468 measured reflections	*l* = −14→15
5342 independent reflections	

### Refinement

**Table:**

Refinement on *F*^2^	Primary atom site location: structure-invariant direct methods
Least-squares matrix: full	Secondary atom site location: difference Fourier map
*R*[*F*^2^ > 2σ(*F*^2^)] = 0.038	Hydrogen site location: inferred from neighbouring sites
*wR*(*F*^2^) = 0.097	H-atom parameters constrained
*S* = 0.95	*w* = 1/[σ^2^(*F*_o_^2^) + (0.0469*P*)^2^] where *P* = (*F*_o_^2^ + 2*F*_c_^2^)/3
5342 reflections	(Δ/σ)_max_ = 0.001
208 parameters	Δρ_max_ = 0.67 e Å^−^^3^
0 restraints	Δρ_min_ = −0.32 e Å^−^^3^

### Special details

**Table:**

Experimental. The intensity data was collected on a Bruker APEXII CCD diffractometer using a frame width of 0.5° covering up to θ = 32.06° with 99.8% completeness accomplished.Spectroscopic data for the flavan ligand: ^1^H NMR (CDCl_3_, 600 MHz) δ p.p.m. 7.46 – 7.30 (5*H*, m, Ar—H), 7.21 – 7.10 (2*H*, m, Ar—H), 6.94 – 6.86 (2*H*, m, Ar—H), 5.08 (1*H*, dd, J = 2.45, 9.98 Hz), 3.02 (1*H*, ddd, J = 6.03, 11.02, 16.46 Hz), 2.81 (1*H*, ddd, J = 3.58, 4.71, 16.46 Hz), 2.27 – 2.04 (2*H*, m);^13^C NMR (600 MHz, CDCl_3_) δ p.p.m. 25.18, 30.06, 77.82, 117.04, 120.43, 121.93, 126.10, 127.45, 127.91, 128.61, 129.63, 141.87, 155.25.Spectroscopic data for the title compound, tricarbonyl(A-η^6^flavan)chromium(0): Note: A, B and C-ring labelling refers to the benzene, phenyl and dihydropyrane rings respectively. ^1^H NMR (600 MHz, CDCl_3_) δ p.p.m. 7.46 (2*H*, d, J = 7.30 Hz), 7.40 (2*H*, dd, J = 7.24, 7.30 Hz), 7.37 – 7.35 (1*H*, m), 5.56 (1*H*, d, J = 6.03 Hz), 5.48 (1*H*, dd, J = 6.02, 6.69 Hz), 5.20 (1*H*, d, J = 6.69 Hz), 4.90 – 4.88 (2*H*, m), 3.00 (1*H*, ddd, J = 5.27, 12.42, 15.95 Hz), 2.64 (1*H*, dd, J = 4.14, 15.95 Hz), 2.31 (1*H*, ddd, J = 4.14, 12.42, 13.93 Hz), 2.17 (1*H*, dd, J = 5.27, 13.93 Hz);^13^C NMR (600 MHz, CDCl_3_) δ p.p.m. 25.94 (C-4), 29.67 (C-3), 80.00 (C-2/6/8), 80.29 (C-2/6/8), 85.87 (C-2/6/8), 94.50 (C-5/7), 95.60 (C-5/7), 126.49 (C-2' and C-6'), 128.80, 128.89, 139.71, 234.06 (–Cr(CO)3);MS m/*z* 346 (*M*+, 39.0), 290 (2.7), 263 (22.1), 222 (56.9), 193 (12.0), 167 (100.0), 158 (99.8), 149 (68.9),129 (1.9), 127 (13.0), 121 (27.0), 106 (31.3), 104 (4.8), 103 (7.8).

### Fractional atomic coordinates and isotropic or equivalent isotropic displacement parameters (Å^2^)

**Table:**

	*x*	*y*	*z*	*U*_iso_*/*U*_eq_	
C1'	0.42382 (14)	0.18308 (12)	0.19968 (17)	0.0245 (3)	
C2	0.50936 (14)	0.20966 (13)	0.12917 (17)	0.0263 (3)	
H2	0.4643	0.2420	0.0405	0.032*	
C2'	0.32347 (17)	0.24327 (15)	0.1760 (2)	0.0372 (4)	
H2'	0.3095	0.2986	0.1167	0.045*	
C3	0.60863 (15)	0.27990 (13)	0.21249 (18)	0.0293 (4)	
H3A	0.6526	0.2491	0.3013	0.035*	
H3B	0.5746	0.3433	0.2285	0.035*	
C3'	0.24388 (17)	0.22168 (16)	0.2401 (2)	0.0405 (5)	
H3'	0.1772	0.2629	0.2240	0.049*	
C4	0.69396 (15)	0.30169 (12)	0.13848 (18)	0.0269 (3)	
H4A	0.6552	0.3457	0.0600	0.032*	
H4B	0.7645	0.3364	0.2005	0.032*	
C4'	0.26295 (15)	0.13974 (15)	0.32735 (18)	0.0327 (4)	
H4'	0.2084	0.1245	0.3686	0.039*	
C5'	0.36356 (16)	0.08021 (13)	0.35341 (18)	0.0295 (4)	
H5'	0.3773	0.0254	0.4135	0.035*	
C5	0.83325 (14)	0.19620 (13)	0.05541 (16)	0.0261 (3)	
H5	0.8817	0.2531	0.0654	0.031*	
C6'	0.44412 (14)	0.10155 (12)	0.29068 (18)	0.0260 (3)	
H6'	0.5119	0.0614	0.3094	0.031*	
C6	0.86366 (15)	0.10536 (13)	0.00632 (17)	0.0290 (4)	
H6	0.9293	0.1028	−0.0204	0.035*	
C7	0.79393 (15)	0.01836 (14)	−0.00212 (18)	0.0309 (4)	
H7	0.8147	−0.0427	−0.0322	0.037*	
C8	0.69312 (14)	0.02274 (13)	0.03445 (17)	0.0278 (4)	
H8	0.6488	−0.0357	0.0319	0.033*	
C9	0.65942 (13)	0.11637 (12)	0.07510 (16)	0.0223 (3)	
C10	0.72998 (13)	0.20345 (12)	0.09035 (15)	0.0219 (3)	
C11	0.79274 (15)	0.03598 (13)	0.34020 (18)	0.0297 (4)	
C12	0.96333 (16)	−0.02379 (14)	0.26452 (19)	0.0334 (4)	
C13	0.95879 (14)	0.16059 (13)	0.34028 (17)	0.0267 (3)	
O1	0.75227 (13)	0.00961 (11)	0.41970 (15)	0.0468 (4)	
O2	1.03289 (14)	−0.08866 (11)	0.29813 (18)	0.0576 (4)	
O3	1.02610 (11)	0.21213 (11)	0.42255 (14)	0.0422 (3)	
O5	0.55780 (9)	0.11377 (8)	0.10382 (12)	0.0260 (2)	
Cr	0.85184 (2)	0.078021 (19)	0.21024 (3)	0.02026 (7)	

### Atomic displacement parameters (Å^2^)

**Table:**

	*U*^11^	*U*^22^	*U*^33^	*U*^12^	*U*^13^	*U*^23^
C1'	0.0243 (7)	0.0262 (8)	0.0257 (8)	−0.0011 (6)	0.0126 (6)	−0.0017 (6)
C2	0.0242 (8)	0.0296 (8)	0.0261 (8)	0.0011 (6)	0.0106 (6)	0.0006 (7)
C2'	0.0396 (10)	0.0401 (10)	0.0380 (10)	0.0150 (8)	0.0215 (8)	0.0135 (8)
C3	0.0306 (9)	0.0270 (8)	0.0338 (9)	−0.0040 (6)	0.0160 (7)	−0.0058 (7)
C3'	0.0318 (9)	0.0562 (12)	0.0377 (10)	0.0187 (9)	0.0179 (8)	0.0066 (9)
C4	0.0286 (8)	0.0225 (7)	0.0312 (9)	−0.0040 (6)	0.0131 (7)	0.0017 (7)
C4'	0.0264 (8)	0.0472 (11)	0.0288 (9)	−0.0049 (7)	0.0152 (7)	−0.0042 (8)
C5'	0.0336 (9)	0.0287 (8)	0.0288 (9)	−0.0049 (7)	0.0147 (7)	0.0016 (7)
C5	0.0237 (8)	0.0346 (9)	0.0218 (8)	−0.0047 (6)	0.0105 (6)	0.0040 (7)
C6'	0.0226 (8)	0.0257 (8)	0.0296 (9)	0.0014 (6)	0.0094 (6)	−0.0017 (7)
C6	0.0251 (8)	0.0436 (10)	0.0229 (8)	−0.0020 (7)	0.0140 (6)	−0.0025 (7)
C7	0.0283 (8)	0.0389 (10)	0.0277 (9)	−0.0027 (7)	0.0131 (7)	−0.0133 (8)
C8	0.0247 (8)	0.0316 (9)	0.0283 (8)	−0.0079 (6)	0.0112 (7)	−0.0119 (7)
C9	0.0182 (7)	0.0304 (8)	0.0189 (7)	−0.0032 (6)	0.0075 (6)	−0.0037 (6)
C10	0.0212 (7)	0.0267 (8)	0.0181 (7)	−0.0016 (6)	0.0076 (6)	0.0020 (6)
C11	0.0268 (8)	0.0330 (9)	0.0288 (9)	0.0007 (7)	0.0096 (7)	0.0045 (7)
C12	0.0334 (9)	0.0314 (9)	0.0372 (10)	0.0039 (7)	0.0150 (8)	−0.0010 (8)
C13	0.0208 (7)	0.0347 (9)	0.0263 (8)	0.0028 (6)	0.0106 (6)	−0.0032 (7)
O1	0.0496 (9)	0.0583 (9)	0.0418 (8)	0.0003 (7)	0.0278 (7)	0.0146 (7)
O2	0.0532 (10)	0.0460 (9)	0.0714 (12)	0.0253 (7)	0.0206 (9)	0.0070 (8)
O3	0.0290 (7)	0.0562 (8)	0.0387 (7)	−0.0062 (6)	0.0091 (6)	−0.0196 (7)
O5	0.0200 (5)	0.0275 (6)	0.0347 (6)	−0.0051 (4)	0.0149 (5)	−0.0083 (5)
Cr	0.01890 (12)	0.02262 (13)	0.02079 (13)	−0.00077 (9)	0.00912 (9)	−0.00132 (10)

### Geometric parameters (Å, °)

**Table:**

C1'—C2'	1.387 (2)	C5—H5	0.9300
C1'—C6'	1.393 (2)	C6'—H6'	0.9300
C1'—C2	1.511 (2)	C6—C7	1.401 (2)
C2—O5	1.4535 (19)	C6—H6	0.9300
C2—C3	1.506 (2)	C7—C8	1.401 (2)
C2—H2	0.9800	C7—H7	0.9300
C2'—C3'	1.385 (3)	C8—C9	1.410 (2)
C2'—H2'	0.9300	C8—H8	0.9300
C3—C4	1.523 (2)	C9—O5	1.3627 (18)
C3—H3A	0.9700	C9—C10	1.399 (2)
C3—H3B	0.9700	C11—O1	1.160 (2)
C3'—C4'	1.374 (3)	C12—O2	1.155 (2)
C3'—H3'	0.9300	C13—O3	1.157 (2)
C4—C10	1.506 (2)	C5—Cr	2.1936 (18)
C4—H4A	0.9700	C6—Cr	2.2163 (19)
C4—H4B	0.9700	C7—Cr	2.2087 (19)
C4'—C5'	1.381 (2)	C8—Cr	2.2251 (17)
C4'—H4'	0.9300	C9—Cr	2.2773 (17)
C5'—C6'	1.384 (2)	C10—Cr	2.2532 (17)
C5'—H5'	0.9300	C11—Cr	1.8353 (19)
C5—C6	1.401 (2)	C12—Cr	1.8298 (19)
C5—C10	1.421 (2)	C13—Cr	1.8399 (17)
			
C2'—C1'—C6'	118.79 (15)	C9—C8—Cr	73.78 (9)
C2'—C1'—C2	119.07 (15)	C7—C8—H8	120.2
C6'—C1'—C2	122.11 (14)	C9—C8—H8	120.2
O5—C2—C3	110.59 (13)	Cr—C8—H8	127.0
O5—C2—C1'	106.22 (13)	O5—C9—C10	123.47 (14)
C3—C2—C1'	113.41 (14)	O5—C9—C8	115.20 (14)
O5—C2—H2	108.8	C10—C9—C8	121.26 (14)
C3—C2—H2	108.8	O5—C9—Cr	130.29 (11)
C1'—C2—H2	108.8	C10—C9—Cr	71.08 (9)
C3'—C2'—C1'	120.57 (17)	C8—C9—Cr	69.75 (9)
C3'—C2'—H2'	119.7	C9—C10—C5	117.84 (15)
C1'—C2'—H2'	119.7	C9—C10—C4	120.15 (13)
C2—C3—C4	111.16 (14)	C5—C10—C4	121.99 (14)
C2—C3—H3A	109.4	C9—C10—Cr	72.96 (9)
C4—C3—H3A	109.4	C5—C10—Cr	69.09 (9)
C2—C3—H3B	109.4	C4—C10—Cr	130.54 (11)
C4—C3—H3B	109.4	O1—C11—Cr	178.15 (15)
H3A—C3—H3B	108.0	O2—C12—Cr	179.38 (19)
C4'—C3'—C2'	120.36 (17)	O3—C13—Cr	179.64 (17)
C4'—C3'—H3'	119.8	C9—O5—C2	118.10 (12)
C2'—C3'—H3'	119.8	C12—Cr—C11	89.46 (8)
C10—C4—C3	109.83 (13)	C12—Cr—C13	88.32 (8)
C10—C4—H4A	109.7	C11—Cr—C13	89.30 (8)
C3—C4—H4A	109.7	C12—Cr—C5	127.56 (7)
C10—C4—H4B	109.7	C11—Cr—C5	142.85 (7)
C3—C4—H4B	109.7	C13—Cr—C5	88.82 (8)
H4A—C4—H4B	108.2	C12—Cr—C7	88.52 (8)
C3'—C4'—C5'	119.60 (16)	C11—Cr—C7	124.86 (8)
C3'—C4'—H4'	120.2	C13—Cr—C7	145.65 (7)
C5'—C4'—H4'	120.2	C5—Cr—C7	66.58 (7)
C4'—C5'—C6'	120.51 (16)	C12—Cr—C6	96.67 (8)
C4'—C5'—H5'	119.7	C11—Cr—C6	160.06 (7)
C6'—C5'—H5'	119.7	C13—Cr—C6	109.76 (7)
C6—C5—C10	121.51 (15)	C5—Cr—C6	37.03 (6)
C6—C5—Cr	72.37 (10)	C7—Cr—C6	36.93 (7)
C10—C5—Cr	73.65 (9)	C12—Cr—C8	108.88 (8)
C6—C5—H5	119.2	C11—Cr—C8	93.63 (8)
C10—C5—H5	119.2	C13—Cr—C8	162.56 (7)
Cr—C5—H5	126.7	C5—Cr—C8	78.49 (7)
C5'—C6'—C1'	120.15 (15)	C7—Cr—C8	36.83 (6)
C5'—C6'—H6'	119.9	C6—Cr—C8	66.43 (7)
C1'—C6'—H6'	119.9	C12—Cr—C10	163.51 (7)
C5—C6—C7	119.17 (15)	C11—Cr—C10	106.33 (7)
C5—C6—Cr	70.60 (9)	C13—Cr—C10	96.43 (7)
C7—C6—Cr	71.24 (9)	C5—Cr—C10	37.25 (6)
C5—C6—H6	120.4	C7—Cr—C10	78.64 (7)
C7—C6—H6	120.4	C6—Cr—C10	66.85 (6)
Cr—C6—H6	130.2	C8—Cr—C10	66.24 (6)
C8—C7—C6	120.50 (16)	C12—Cr—C9	144.51 (7)
C8—C7—Cr	72.22 (10)	C11—Cr—C9	86.69 (7)
C6—C7—Cr	71.83 (10)	C13—Cr—C9	126.85 (7)
C8—C7—H7	119.8	C5—Cr—C9	65.36 (6)
C6—C7—H7	119.8	C7—Cr—C9	65.51 (6)
Cr—C7—H7	128.5	C6—Cr—C9	77.30 (6)
C7—C8—C9	119.50 (15)	C8—Cr—C9	36.47 (6)
C7—C8—Cr	70.95 (10)	C10—Cr—C9	35.96 (5)

## References

[bb1] Altomare, A., Burla, M. C., Camalli, M., Cascarano, G. L., Giacovazzo, C., Guagliardi, A., Moliterni, A. G. G., Polidori, G. & Spagna, R. (1999). *J. Appl. Cryst.* **32**, 115–119.

[bb2] Brandenburg, K. & Putz, H. (1999). *DIAMOND* Crystal Impact GbR, Bonn, Germany.

[bb3] Bruker (2001). *SADABS* Bruker AXS Inc., Madison, Wisconsin, USA.

[bb4] Bruker (2007). *APEX2* and *SAINT-Plus* Bruker AXS Inc., Madison, Wisconsin, USA.

[bb5] Farrugia, L. J. (1999). *J. Appl. Cryst.* **32**, 837–838.

[bb6] Müller, T. J. J., Ansorge, M. & Polburn, K. (1999). *J. Organomet. Chem.* **578**, 252–259.

[bb7] Muschalek, B., Weidner, I. & Butenschön, H. (2007). *J. Organomet. Chem.* **692**, 2415–2424.

[bb8] Sheldrick, G. M. (2008). *Acta Cryst.* A**64**, 112–122. 10.1107/S0108767307043930 18156677

[bb9] Tonder, J. H. van, Bezuidenhoudt, B. C. B. & Janse van Rensburg, J. M. (2009*a*). *Acta Cryst.* E**65**, m1343. 10.1107/S1600536809040537 PMC2971219 21578099

[bb10] Tonder, J. H. van, Bezuidenhoudt, B. C. B. & Janse van Rensburg, J. M. (2009*b*). *Acta Cryst.* E**65**, m1346. 10.1107/S1600536809040525 PMC2971426 21578102

